# Exploring molecular spectrum in thai patients with maple syrup urine disease: unveiling a common variant

**DOI:** 10.1186/s13023-024-03411-7

**Published:** 2024-10-25

**Authors:** Panisara Lakkhana, Thipwimol Tim-Aroon, Arthaporn Khongkraparn, Saisuda Noojarern, Parith Wongkittichote, Khunton Wichajarn, Chulaluck Kuptanon, Boonchai Boonyawat, Kanya Suphapeetiporn, Karn Wejaphikul, GoHun Seo, Duangrurdee Wattanasirichaigoon

**Affiliations:** 1 Department of Pediatrics, Faculty of Medicine Ramathibodi Hospital, Mahidol University, Bangkok, Thailand; 2 Division of Medical Genetics, Department of Pediatrics, Faculty of Medicine Ramathibodi Hospital, Mahidol University, Bangkok, Thailand; 3 Division of Epidemiology and Translational Research, Department of Pediatrics, Faculty of Medicine Ramathibodi Hospital, Mahidol University, Bangkok, Thailand; 4 Division of Medical Genetics, Department of Pediatrics, Srinagarind Hospital, Khon Kaen, Thailand; 5 Division of Medical Genetics, Department of Pediatrics, Queen Sirikit National Institute of Child Health, Bangkok, Thailand; 6 Division of Medical Genetics, Department of Pediatrics, Phramongkutklao Hospital, Bangkok, Thailand; 7 Department of Pediatrics, Faculty of Medicine, Excellence Center for Genomics and Precision Medicine, King Chulalongkorn Memorial Hospital, Thai Red Cross Society, Chulalongkorn University, Bangkok, Thailand; 8 Department of Pediatrics, Faculty of Medicine, Chiang Mai University, Chiang Mai, Thailand; 9 Division of Medical Genetics, 3Billion Inc., Seoul, South Korea

**Keywords:** Maple syrup urine disease, MSUD, Branched-chain alpha-keto dehydrogenase complex, *BCKDHB*, Inborn errors of metabolism, Genotypic spectrum

## Abstract

**Background:**

Maple syrup urine disease (MSUD) is a rare autosomal recessive metabolic disorder caused by variants in any of the following genes: *BCKDHA*, *BCKDHB,* and *DBT* gene. Previous reports have highlighted a variety of common causing genes and variants among different ethnic groups affected by MSUD. This study is the first to describe the molecular characteristics, potential common variants, clinical phenotypes, and treatment outcomes of 20 Thai MSUD patients before the implementation of expanded newborn screening in Thailand.

**Results:**

A cross-sectional, multicenter study was conducted, including twenty Thai MSUD patients from 1997 to 2023. Most of the patients presented with classic neonatal onset (95%). The mortality rate was 20%, while global developmental delay was observed in 40% of the patients. Variants in the *BCKDHB* gene were detected in 85% (17/20) of the patients, while the *BCKDHA* gene accounted for 15% (3/20). The study identified the 11-kb deletion involving 5’UTR, exon 1, and intron 1 in the *BCKDHB* gene, from a position of g.80102385 to g.80113453 (NC_000006.12), accounting for 50% of all variants (20/40 alleles) in Thai MSUD patients. All patients with the 11-kb deletion in *BCKDHB* presented with the classic type. The gap-PCR for this common deletion was established in the study.

**Conclusion:**

This study is the first to describe the clinical and molecular spectrum of Thai MSUD patients before the implementation of expanded NBS. The 11-kb deletion involving exon 1 in the *BCKDHB* emerges as the most common variant among Thai individuals with MSUD. Furthermore, the gap-PCR test for detecting the 11-kb exon 1 deletion status holds the potential for integration into stepwise molecular analysis following positive expanded newborn screening.

**Supplementary Information:**

The online version contains supplementary material available at 10.1186/s13023-024-03411-7.

## Background

Maple syrup urine disease (MSUD, MIM #248600) is an autosomal recessive inherited metabolic disorder caused by the deficiency of branched-chain α-ketoacid dehydrogenase (BCKDH) complex, an enzyme responsible for the second step of branched-chain amino acids catabolism [1, 2]. The incidence of MSUD varies across different ethnicities, with an overall reported rate of 1 in 185,000 live births. [2] For instance, Mennonite populations exhibit a notably higher incidence, estimated at 1 in 380 live births. In Spain, the incidence ranges between 1 in 12,000–50,000 live births, while in Portugal, it is reported to be approximately 1 in 86,800 live births. [2–5]

Clinical presentations of MSUD include feeding difficulties, epilepsy, intellectual disability, ketonuria, and a maple syrup-like odor. [1, 2] Biallelic pathogenic variants in *BCKDHA, BCKDHB*, and *DBT* genes have been found to be associated with MSUD, with reported proportions of 45%, 35%, and 20%, respectively [2]. Notably, the distribution of disease-causing genes varies across different countries, underscoring the complex genetic landscape of MSUD [2–10].

While clinical and biochemical assessments are crucial for the initial diagnosis, genetic testing plays an indispensable role in confirming diagnoses, providing genetic counseling, and achieving a comprehensive understanding of molecular epidemiology. In Thailand, the incidence of MSUD remains undetermined. Expanded newborn screening, including MSUD, was initiated in October 2022. Prior to this, most of the patients were diagnosed based on the presence of clinical symptoms. Limited literature, comprising four scientific publications detailing 17 Thai MSUD patients have been reported without an exploration of genetic spectrum. [11–14] This underscores the need for further investigation into the genetic landscape of the disorder within this population.

In this study, we delineated a genetic spectrum and proposed a common variant among MSUD patients in Thailand. Additionally, we described clinical findings and outcomes of Thai patients with MSUD prior to the implementation of expanded NBS.

## Methods

### Patients

This multicenter, cross-sectional study was conducted including patients from January 1997 to November 2023. Participants were diagnosed based on clinical symptoms and biochemical testing, with or without genetic test results. Thai MSUD patients were included from the five Rare Disease Centers, including Ramathibodi Hospital, Srinagarind Hospital, Queen Sirikit Institute of Child Health, King Chulalongkorn Memorial Hospital, and Phramongkutklao Hospital. Patients lacking clinical symptom data or available genetic test results or DNA specimens were excluded. Individuals who either declined enrollment or withdrew from the study were excluded from the analysis. The study was approved by the Human Research Ethics Committee, Faculty of Medicine Ramathibodi Hospital, Mahidol University (COA: MURA2022/423 Ref.3493).

### Clinical analysis

Demographic and clinical data, encompassing gender, hometown, family history, parental consanguinity, initial clinical presentations, laboratory investigations, treatment modalities, and outcomes during the study period, were collected for analysis in this study.

### Molecular analysis

Genomic DNA was extracted from peripheral blood specimens following standard protocols (QIAGEN GmbH). Clinical exome sequencing with targeted analysis focusing on three disease-causing genes (*BCKDHA, BCKDHB*, and *DBT)* and/or Sanger sequencing were initially performed in all patients. Databases used for determining possible pathogenicity and minor allele frequency (MAF) included ClinVar (https://www.ncbi.nlm.nih.gov/clinvar), the Human Genome Mutation Database (http://www.biobase-international.com), and population databases including the gnomAD, dbSNP version 142, 1000Genome phase3, the Exome Variant Server (version ESP6500SI V2; https://evs.gs.washington.edu/EVS/), the T-REx database (Thai genome database of 2000 exome sequencing, https://trex.nbt.or.th/), and our in-house whole exome database of 300. To determine a possible deleterious effect of a variant identified at the splicing region, NNSplice (https://www.fruitfly.org/seq_tools/splice.html) was employed.

**Fig. 1:**
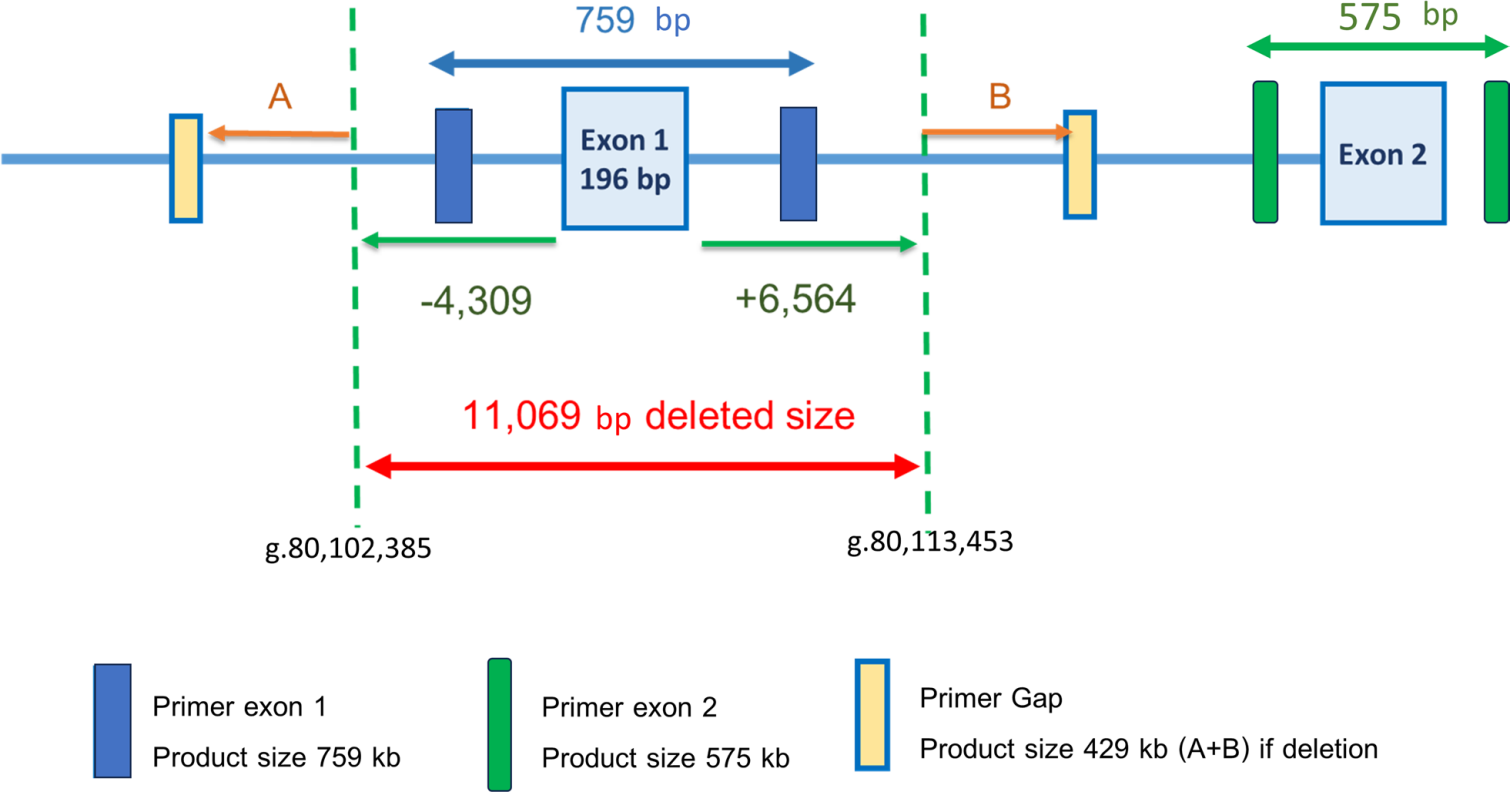
Position of the 11-kb deleted region in *BCKDHB* and the gap-PCR primers with the product sizes. This figure shows the 11-kb deleted region between g.80,102,385 and g.80,113,453 (A red arrow). Primers of exon 1 (blue boxes) with a product size of 759 bp, primers of exon 2 (green boxes) with a product size of 575 bp, and primers of a gap for the deleted region (yellow boxes) with a product size of 429 bp (A + B) if having the 11-kb deletion

Disease-causing variants were classified based on the 2015 guidelines of the American College of Medical Genetics and Genomics and the Association of Molecular Pathology (ACMG/AMP) for sequence variants and the 2020 guidelines of the American College of Medical Genetics and Clinical Genome Resource (ClinGen) for copy number variants. [15, 16] To determine the precise position and size of the exon 1 deletion in *BCKDHB* gene, whole genome sequencing (WGS) was performed in the two MSUD patients (patient 4 and 5) who were initially not found to have biallelic variants by clinical exome sequencing. The result of WGS was aimed to determine the precise position and size of the deleted region. Gap-PCR technique was developed to detect the 11-kb deletion of the *BCKDHB.* PCR primers for each product were designed as supplement data (Table S1). The gap-PCR for 11-kb exon 1 deletion of *BCKDHB* was successfully developed under a proper condition. Three distinct components: 1) exon 1 product (759 bp), 2) exon 2 product (575 bp) serving as a control, and 3) Gap 11-kb exon 1 deletion (429 bp) as shown in Fig. 1 were used to distinguish wild type, heterozygotes, and homozygotes of this specific variant. PCR primers for each product were designed as supplement data (Table S1). The newly developed gap-PCR test was performed on the MSUD patients who lacked a definitive molecular diagnosis from sequencing.

### Statistical analysis

Descriptive and proportion analysis were conducted, supplemented by logistic regression to assess the effect of genotype-phenotypic correlation.

## Results

### Clinical characteristics

The study recruited 30 unrelated individuals with MSUD from the five rare disease centers. Ten patients were excluded due to insufficient clinical information or unknown genetic test results with unavailable DNA samples. Subsequently, a total of 20 unrelated patients were finally included in the study. The patients were distributed across various regions, with 40% located in the Central region, 40% in the North-Eastern region, 10% in the Northern region, 5% in the Eastern region, and 5% in the Southern region of Thailand. Parental consanguinity was reported in 5 patients (25%).

Comprehensive patient profiles, encompassing gender, age of onset, age of diagnosis, clinical outcomes, and treatment modalities, are delineated in Table 1. Among the 20 enrolled patients, 65% (13/20) were males and 35% (7/20) were females. All participants were of Thai descent, with the majority clinically diagnosed with the classic neonatal onset form (95%). Only one patient (Patient 17) exhibited an intermediate type. Symptoms and signs during the first admission included poor feeding (100%), hyperammonemia (87.5%), convulsions (85%), maple syrup-like odor (75%), metabolic acidosis (70%), encephalopathy (65%), respiratory failure (55%), and hypoglycemia (50%). The median age of onset was 7 days (range: 4–90 days), while the median age of diagnosis was 16 days (range: 11–365 days).

**Table 1:** Clinical and molecular characterization of the patients in this study

Patient ID	Gender	Age of onset (days)	Age of diagnosis (days)	Consan-guinity	Total blood exchange	Hemo/peritoneal dialysis	Liver transplantation (age at LT)	Follow-up outcome	Status (age at study/ age of death)	Plasma Leucine (μmol/L)	Gene	Variant 1		Variant 2
1	M	7	47	No	No	No	No	DD (mild-mod), epilepsy	Alive (3 y 8 m)	2,605	*BCKDHB*		11-kb exon1 deletion	11-kb exon1 deletion
2	F	9	11	No	Yes	No	No	DD (severe), bed-ridden, epilepsy	Dead (2 m)	5,146	*BCKDHB*		11-kb exon1 deletion	c.730delT (p.Tyr244Thrfs*10)
3	M	4	12	No	No	Yes	No	DD (mild-mod), epilepsy, ADHD	Alive (7 y 4 m)	> 3,000	*BCKDHB*		11-kb exon1 deletion	c.730delT (p.Tyr244Thrfs*10)
4	M	7	60	No	No	No	No	DD (severe), epilepsy, FTT	Alive (26 y)	2,603	*BCKDHB*		11-kb exon1 deletion	c.723A > T (p.Lys241Asn)
5	F	10	16	Yes	Yes	No	No	DD (mild-mod), FTT, ADHD, autistic-like	Alive (13 y)	1,420	*BCKDHB*		11-kb exon1 deletion	11-kb exon1 deletion
6	M	7	13	No	Yes	No	Yes (10 y)	DD (mild-mod), FTT, epilepsy, hearing impairment	Alive (10 y 9 m)	> 3,000	*BCKDHB*		11-kb exon1 deletion	c.1032_1033insTA (p.Val345*)
7	M	5	30	Yes	No	Yes	Yes (1 y 9 m)	DD (mild-mod), FTT, epilepsy	Alive (10 y 2 m)	> 3,000	*BCKDHA*		c.511delC (p.Leu171Trpfs*159)	c.511delC (p.Leu171Trpfs*159)
8	F	7	21	No	No	No	No	DD (mild-mod), epilepsy	Dead (19 y)	2,300	*BCKDHB*		11-kb exon1 deletion	11-kb exon1 deletion
9	M	10	14	No	No	No	No	DD (mild-mod)	Alive (10 y 7 m)	N/A	*BCKDHB*		c.444G > C (p.Gln148His)	c.444G > C (p.Gln148His)
10	M	10	12	No	No	Yes	No	DD (mild-mod)	Alive (1 y 2 m)	2,000	*BCKDHB*		11-kb exon1 deletion	11-kb exon1 deletion
11	M	7	16	No	No	No	Yes (1 y)	Normal development	Alive (3 y)	N/A	*BCKDHA*		c.859C > T (p.Arg287*)	c.1145G > A (p.Try382*)
12	M	14	60	No	No	No	No	DD (severe), FTT, microcephaly	Alive (8 y 6 m)	3,611	*BCKDHB*		c.730delT (p.Tyr244Thrfs*10)	c.730delT (p.Tyr244Thrfs*10)
13	F	5	47	No	No	No	No	DD (severe), FTT, epilepsy, microcephaly	Alive (16 y)	3,149	*BCKDHB*		11-kb exon1 deletion	11-kb exon1 deletion
14	M	10	22	Yes	No	No	No	DD (severe), FTT, epilepsy, microcephaly	Alive (9 y 3 m)	1,761	*BCKDHB*		11-kb exon1 deletion	11-kb exon1 deletion
15	F	7	14	No	No	Yes	No	Normal development	Alive (1 y 3 m)	4,503	*BCKDHB*		11-kb exon1 deletion	11-kb exon1 deletion
16	F	7	15	No	No	Yes	Yes (11 m)	DD (mild-mod)	Alive (2 y 9 m)	2,923	*BCKDHB*		11-kb exon1 deletion	c.403G > A (p.Gly135Arg)
17	F	90	365	No	No	No	No	DD (severe), epilepsy	Alive (10 y)	2,115	*BCKDHB*		c.365C > T (p.Thr122Ile)	c.1096C > T (p.Pro366Ser)
18	M	10	13	Yes	Yes	Yes	No	DD (severe), epilepsy	Dead (1 y 2 m)	N/A	*BCKDHA*		c.629C > T (p.Ala210Val)	c.647C > T (p.Ala216Val)
19	M	15	20	Yes	Yes	No	No	DD (severe), FTT epilepsy	Dead (1 y 7 m)	N/A	*BCKDHB*		c.547C > T (p.Arg183Trp)	c.547C > T (p.Arg183Trp)
20	M	5	12	No	Yes	Yes	No	Mild gross motor delay	Alive (4 m)	1,250	*BCKDHB*		11-kb exon1 deletion	c.506A > G (p.Tyr169Cys)

ADHD, attention deficit hyperactivity disorder; DD, developmental delay; F, female; FTT, failure to thrive; LT, liver transplantation; M, male; m, months; N/A, not applicable; y, years

Branched-chain amino acid restriction with close monitoring of amino acid concentrations was the mainstay treatment in all patients. Additional interventions include hemo/peritoneal dialysis (35%), total blood exchange (30%), and liver transplantation (20%). Among a total of 20 patients, 4 (20%) patients passed away, while 16 (80%) patients remained alive. Notably, an individual expired before the age of 2 months. Among the nineteen patients who survived beyond 2 months of age, 60% had epilepsy and 90% exhibited global developmental delay (GDD), with 40% experiencing severe GDD.

### Molecular spectrum

**Fig. 2:**
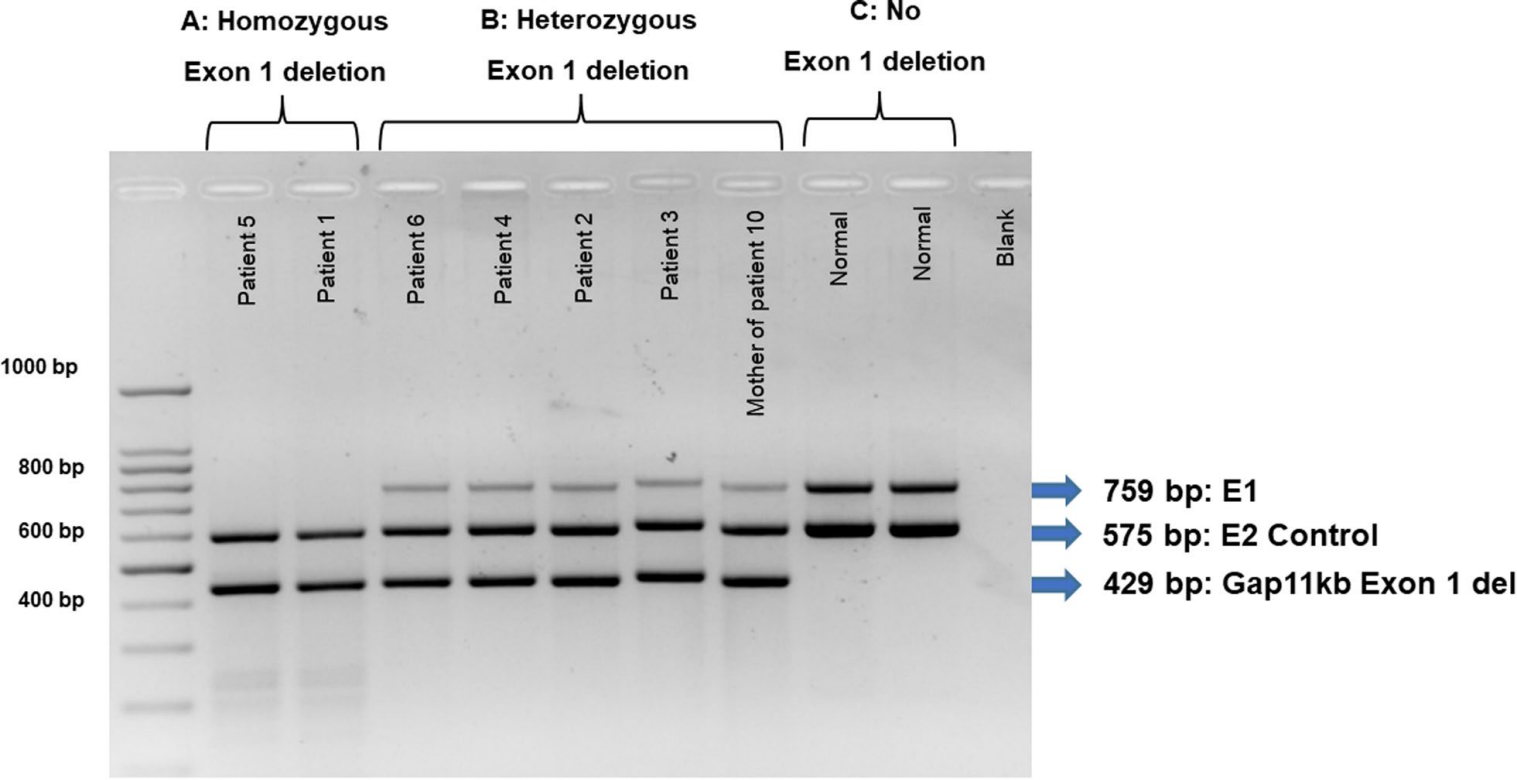
The gap-PCR result for the 11-kb deletion in the *BCKDHB*. **A** The results of homozygous 11-kb deletion show 2 bands (575 and 429 bp) in patient 1 and 5; **B** The results of heterozygous 11-kb deletion show 3 bands (759, 575 and 429 bp) in the patient 2, 3, 4, 6 and mother of patient 10; **C** The results of no 11-kb deletion show 2 bands (759 and 575 bp) in two normal controls

The WGS revealed (GrCh38, NC_000006.12) 11.06 kb deletion at a genomic position of g.80102385–g.80113453 of chromosome 6q14.1 in heterozygous and homozygous states in patient 4 and 5, respectively. The deleted region involved the 5’UTR, exon 1, and intron 1 of the *BCKDHB* gene (NM_183050.4) (Fig. 1). The gap-PCR technique successfully detected the 11-kb exon 1 deletion spanning from 5’UTR to intron 1 in *BCKDHB*. This provided a specific tool for identifying homozygous, heterozygous, and wild types, as shown in Fig. 2.

The molecular spectrum of 20 Thai patients is presented in Table 1. Biallelic variants in the *BCKDHB* gene were identified in 17 patients (85%), while three individuals (15%) harbored biallelic variants in the *BCKHDA* gene. The 11-kb exon 1 deletion in *BCKDHB* is the most prevalent variant in the study, accounting for 50% of all variants (20 in 40 alleles) or 58.8% of the *BCKDHB* variants (20 in 34 alleles). The second common variant identified in *BCKDHB* was c.730delT (12%, 4 in 34 alleles). The frequency of each variant is summarized in Table 2. The presentation, plasma leucine concentration, and clinical outcome, including rates of epilepsy, failure to thrive, severe developmental delay, and mortality, did not differ between individuals with the exon 1 deletion in *BCKDHB* and those with other variants*.*

**Table 2:** Distribution of variants and genes in twenty Thai MSUD patients

Gene	Variant	Protein prediction	Reference number	Classification	Frequency
*BCKDHB* NM_183050.4	11-kb deletion involving exon 1	p.Met1?	novel	Pathogenic	50% (20/40)
	c.730delT	p.Tyr244Thrfs*10	rs1057516572	P (PVS1, PM2, PP5)	10% (4/40)
	c.547C>T	p.Arg183Trp	rs149766077	P (PM1, PM2, PM5, PP2, PP3, PP5)	5% (2/40)
	c.444G>C	p.Gln148His	novel	LP (PM2, PP3, PP2, PP4)	5% (2/40)
	c.1032_1033insTA	p.Val345*	novel	P (PVS1, PM2, PM3)	2.5% (1/40)
	c.506A>G	(p.Tyr169Cys)	rs398124580	P (PM1, PM2, PM3, PP2, PP3)	2.5% (1/40)
	c.723A>T	p.Lys241Asn	novel	LP (PM2, PM3, PP2, PP3)	2.5% (1/40)
	c.403G>A	p.Gly135Arg	rs751953459	P (PM2, PM3, PP2, PP3)	2.5% (1/40)
	c.365C>T	p.Thr122Ile	rs398124575	VUS (PM2, PP2, PP3)	2.5% (1/40)
	c.1096C>T	p.Pro366Ser	novel	VUS (PM1, PM2, PP2, PP3)	2.5% (1/40)
*BCKDHA* NM_000709.4	c.511delC	p.Leu171Trpfs*159	rs762084007	P (PVS1, PM2, PP5)	5% (2/40)
	c.859C>T	p.Arg287*	rs764247545	P (PVS1, PS3, PM2, PM3)	2.5% (1/40)
	c.1145G>A	p.Try382*	novel	LP (PVS1, PM2, PM3)	2.5% (1/40)
	c.629C>T	p.Ala210Val	novel	LP (PM2, PP2, PP3)	2.5% (1/40)
	c.647C>T	p.Ala216Val	rs369448982	P (PM2, PM3, PP2, PP3)	2.5% (1/40)

LP, likely pathogenic; P, pathogenic; VUS, variant of uncertain significance

## Discussion

Here, we describe the genetic spectrum, clinical phenotype, and treatment outcomes in 20 Thai patients with MSUD from 1997 to 2023, while the implementation of expanded newborn screening (NBS) in Thailand was initiated in October 2022. One patient was diagnosed after the initiation of expanded NBS; however, the age of onset was earlier than the age of NBS result. Most patients in this study exhibited symptoms and signs of classic MSUD (95%), a proportion consistent with previous studies, which have reported ranges from 64 to 84%. [5–8] Given this, the clinical symptoms leading to medical attention in this study might differ from those observed in countries with expanded NBS for MSUD. [17–19] An earlier clinical report from Thailand in 2008 demonstrated clinical phenotypes of thirteen Thai classic MSUD patients who were not part of the present study. [12] The report in 2008 indicated a median age of diagnosis of 55 days (range: 18–365 days), which is comparatively later than the present study’s findings, where the median age of diagnosis was 20 days (range: 11–365 days). [12] Neurodevelopmental delay was observed in all patients in the report in 2008, while severe GDD was found in 40% of the patients in the present study. In our cohort, four patients (20%) underwent liver transplantation. Previous literature in the United States and European countries demonstrated that liver transplantation was performed in 46–87% of MSUD patients. [20, 21] The primary reason for the low rate of liver transplantation in Thailand is likely attributable to a limited number of pediatrics liver transplantation centers. The overall mortality rate in this study was 20%, which aligns with findings from previous studies reporting a mortality rate of 37.5% in China, 26.5% in India, and 24% in the Philippines. [8, 19, 22] In light of the potential for late diagnosis to result in long-term disabilities and mortality, it becomes imperative to prioritize early diagnosis and implement appropriate dietary management for achieving favorable outcomes in MSUD patients.

Expanded NBS has been shown to be a cost-effective strategy for improving the quality of life and reducing morbidity and mortality among MSUD patients. [23, 24] Moreover, proper enteral and parenteral treatment protocols have significantly improved neurological outcomes for patients with classic MSUD patient. [1, 22, 25] Other factors, including logistics, referring system, laboratory accessibility, prompt and appropriate treatment, and specialist availability, are necessary. [17, 18, 25] This implies the need for further research and quality improvement to achieve the best possible outcome following the initiation of expanded NBS in Thailand.

This study revealed that the predominant defective gene observed in Thai MSUD patients is the *BCKDHB* gene (85%). This finding differs from global data and multiple countries, where the prevalent variants are commonly reported in *BCKDHA* (45%), followed by *BCKDHB* (35%) and *DBT* (20%). [2] However, previous studies from Egypt, China, Brazil, India, and Malaysia reported a high prevalence of the *BCKDHB* gene with a frequency of 72%, 65%, 57%, 50%, and 44%, respectively [6–10]. This finding underscores the significance of considering regional genetic variations. In the Mennonite population, a founder variant, c.1312T>A (p.Tyr438Asn) in the *BCKDHA* gene (NM_000709.2), is the most common variant. [3] In Malaysia, a founder variant, c.1196C>G (p.Ser399Cys) in the *DBT* gene (NM_001918.4), has been reported. [7]

In our cohort, we identified the 11-kb deletion involving exon 1 spanning from 5’UTR to intron 1 in the *BCKDHB* gene as the most common pathogenic variant. This deletion accounted for 50% of all variants or 59% of variants in the *BCKDHB*. Given the rarity of this deletion in other populations but its prevalence among Thai patients from different regions, the best explanation is the presence of an ancestral allele. We postulate that the emergence of a new common variant in Thai MSUD patients may be attributed to shared ancestry within Southeast Asia, potentially indicative of a founder effect. This is consistent with previous reports for other inborn metabolic diseases in Thailand [26, 27]. However, our study did not conduct a shared identity-by-descent/identity-by-state analysis to confirm the presence of a founder among cases. Furthermore, it is noteworthy that 20% of the included patients have a history of parental consanguinity.

According to literature from Malaysia, the 3.3-kb deletion extending from the 5’UTR to exon 1 (g.80819375–80816718) was documented in Malaysian MSUD patients, observed in 3 out of 31 individuals [7]. The deletion reported in Malaysia differs in location from the deletion identified in the present study. An entire exon 1 deletion has been described in a patient from India (1 out of 24 patients); however, specific details regarding the size or position of the deletion are not available. [8] This present study precisely identified the position of the deleted region (g.80102385–g.80113453), proposing it as a novel variant. We hypothesize that the 11-kb deletion involves the initiation of translation (5’UTR, exon 1, and intron 1), which is expected to produce a truncated protein. Further studies are required to investigate the protein consequence of this 11-kb deletion.

The residual activity of the BCKD enzyme determines the severity of the MSUD phenotype [2]. However, the results of BCKD enzyme activity were not available in the present study. In the absence of enzyme activity results, clinical features and biochemical results are valuable parameters for categorizing individuals into classic or intermediate types. While some studies have reported relationships between genotype and biochemical phenotype (classic or intermediate), clinical outcomes cannot be predicted solely based on genotype. [2, 9] We observed that all patients with the 11-kb exon 1 deletion presented with classic MSUD and significantly increased leucine concentrations. Consistent with the previous reports, the present study did not identify any significant difference in clinical outcomes associated with the common deleted variant or other variants. The major limitation of this study is the small sample size of patients, compounded by the presence of several factors that may influence the outcome.

Since this deletion is the most common variant found in Thai MSUD patients, integrating the gap-PCR for the 11-kb deletion into subsequent steps after positive expanded NBS is warranted. It could also be incorporated into the diagnostic algorithm for patients with abnormal clinical and/or biochemical phenotypes consistent with MSUD. Moreover, this technique might reduce the costs associated with molecular diagnosis and implement carrier testing for the Thai population. However, a prospective study is needed to evaluate its utility and cost-effectiveness.

## Conclusion

We characterized the clinical phenotype and expanded the genotypic spectrum of MSUD, specifically among Thai patients. Identifying the 11-kb deletion involving exon 1 in the *BCKDHB* gene in 50% of MSUD patients highlights the necessity for region-specific genetic insights. The gap-PCR for the 11-kb deletion offers potential integration into stepwise molecular analysis following positive results from expanded newborn screening for Thai MSUD patients.

## Supplementary Information

Additional file 1.

## Data Availability

The data used and/or analyzed in the study are not publicly available due to ethical and privacy considerations to protect the confidentiality of participants but are available from the corresponding author on reasonable request.
