# Supplementary material for: Exploring molecular spectrum in thai patients with maple syrup urine disease: unveiling a common variant
Source: Orphanet J Rare Dis. 2024 Oct 25;19:396. doi: 10.1186/s13023-024-03411-7 (PMC11515341; doi:10.1186/s13023-024-03411-7)
Supplement: Supplementary file 1 — Additional file 1. [file 13023_2024_3411_MOESM1_ESM.pdf]

Supplementation Data

Table S1 Forward and reverse primers for the Gap-PCR detecting the 11-kb exon 1 deletion

Primer name	Forward 5'-3' primer	Reverse 5'-3' primer	PCR product (bp)
BCKDHB E1	AGGCACTGTGTGGAACTTT	TATGCCCTCTCACCCATCT	759
BCKDHB E2	CACGCATTTAGTCACTTGGTTAT	GGACCACACACACTGTAAGA	575
BCKDHB 11kb del	GAGTGGAATTGCTGGGTCATA	CATCCTCCACCAGTAACGTATTT	429
